# Citizens’ Perceptions of Landscape Changes and Their Driving Forces: Evidence from Poland

**DOI:** 10.3390/ijerph19031688

**Published:** 2022-02-01

**Authors:** Iga Solecka, Piotr Krajewski, Aleksandra Krzyżanek, Ada Garczyńska

**Affiliations:** Institute of Spatial Management, Wrocław University of Environmental and Life Sciences, 50-375 Wrocław, Poland; iga.solecka@upwr.edu.pl (I.S.); 113388@student.upwr.edu.pl (A.K.); 113356@student.upwr.edu.pl (A.G.)

**Keywords:** landscape change, driving forces, citizens’ perception, land-use change, landscape change index

## Abstract

The main aim of our studies was to explore the driving forces of landscape change and their impact on the landscape as perceived by citizens in our study. We use quantitative tools for unravelling processes of landscape change over time and a qualitative tool aimed at capturing people’s perceptions about those changes. We use the two municipalities of Ostrów Wielkopolski and Kąty Wrocławskie as illustrative examples of urban and urban–rural municipalities in two time periods, 2006–2021 and 2012–2018, in Poland. We apply a three-stage approach: (1) to identify the main landscape changes based on land-cover data, (2) to characterize those changes with the use of orthophoto maps and (3) to identify the driving forces of landscape changes with the use of an online survey and interviews. The results show a large agreement between the perceived and actual level of changes. We identified key landscape change processes in both municipalities, and we conclude that citizens’ perceptions concerning those processes in both municipalities differed depending on the context, the level of changes, and the way this process was planned and implemented. In both municipalities, the respondents pointed out political driving forces of landscape change as key underlying drivers. Future landscape planning should consider citizens’ approaches towards landscape change to achieve better societal approval and improve the quality of life of the inhabitants.

## 1. Introduction

Landscape change is caused by a complex combination of technological, social, cultural, political and spatial processes [1]. These processes are called drivers [2], or key processes in landscape change [3] or driving forces of landscape change. They are mostly divided into five main groups—socio-economic, political, technological, natural and cultural [4,5]—and are analyzed as proximate and underlying processes [6]. In this paper, we understand proximate processes as human activities or immediate actions at the local level with a direct impact on land-use change (such as agricultural expansion that directly impacts forest cover) and underlying processes as fundamental social processes (such as human population dynamics or agricultural policies) that usually have an indirect but often crucial impact at the national, regional, or global levels, and influence/cause the proximate drivers [6]. In the recent 20 years, an intensification of landscape changes [7] and increasing pressure on landscape values [8] have been observed. Landscape transformations are observed all over the world, especially in the countries of Central and Eastern Europe [9]. The fall of communism was the beginning of landscape transformations in Poland [10]. Another stage of landscape development started in 2004 after Poland’s accession to the European Union and the activation of funds for changing the transport system or the reconstruction of degraded areas [11,12]. Significant changes in the landscape also affected protected areas, e.g., Ślęża Landscape Park in southwestern Poland [13]. Increasingly, the literature emphasizes the need to include social research on the perceptions of citizens in the analyses of the driving forces behind landscape change [14,15].

The way people perceive those changes is helpful to recognize and analyze the change in the social and cultural context [16]. The perceptive approach helps to recognize people’s attitudes and behaviors, which are highly significant direct drivers of local people’s priorities and preferences in the context of landscape changes, and hold important information for planners and decision makers [17]. The perception of landscape change may impact the perceived attractiveness of the living environment which is one of the most important factors affecting residential satisfaction [18,19]. Whether we perceive landscape change as negative or positive may also impact our mood [20] or contribute towards better quality of life [21].

The studies on the perception of landscape change have been focused on the evaluation of the landscape before [22,23] or after the change in landscape (e.g., in forest cover, solar power installations, new housing settlements) [24,25,26]. The perception of landscape change has been researched by semi-structured interviews [26,27,28], surveys/questionnaires [22,25,29], free listings [27] and participatory photography sessions and focus groups [24]. Most of the studies focused on the individual perception of landscape change and their explanatory variables. The study of Twongyirwe et al. [30] about local perceptions of forest cover change shows that age and the type of livelihood are significantly related to the perceptions of forest cover change. The study of Islam et al. [26] about forest co-management in Bangladesh shows that the perception of forest cover change differed between male and female respondents as well as people engaged in forest management and those who were not involved. The perception also depends on what land-cover type is affected by the change. The study of perceptions of landscapes changed by solar power plants shows that on-ground solar power installations were valued differently based on the type of land they occupy [24]. Most of the studies use expert assessment to interpret and identify driving forces [6]; for example, the study in north-eastern Ghana used a mixed method including geospatial analysis, interviews with experts and a questionnaire for researchers and local experts to assess the driving forces of land-use and land-cover change [31]. The perception of landscape change has been widely researched; however, the evidence of how the specific setting (urban and urban–rural landscape) influences the perception of different types of landscape change is still scarce. Strong urbanization and industrialization processes impact urban and rural landscapes all over Europe [32]. Therefore, the motivation for this study arose from the need to recognize how citizens perceive different types of landscape change and what driving forces are associated with this change in two different settings.

The main aim of our studies was to explore the driving forces of landscape change and their impact on the landscape as perceived by citizens. We used quantitative tools for unravelling the processes of landscape change over time and a qualitative tool aimed at capturing people’s perceptions about those changes. We integrated spatial data from the Corine Land Cover database and archival orthophoto maps, and local knowledge derived from social perception studies. Our objectives were synthesized as: (1) quantifying and researching landscape change processes in two case study areas of urban and rural landscapes, (2) recognizing the underlying drivers of landscape change as perceived by the local community and (3) exploring what factors influence the perception of different types of landscape change in different contexts. We formulated two main research questions to which we wanted to find answers:

I. Is the level of change in the landscape across municipalities at a similar level? What factors have influenced this?
II. How are changes in the landscape perceived? Do perceptions of the same types of landscape changes differ in those two municipalities?

## 2. Materials and Methods

### 2.1. Case Study Area

**Figure 1:**
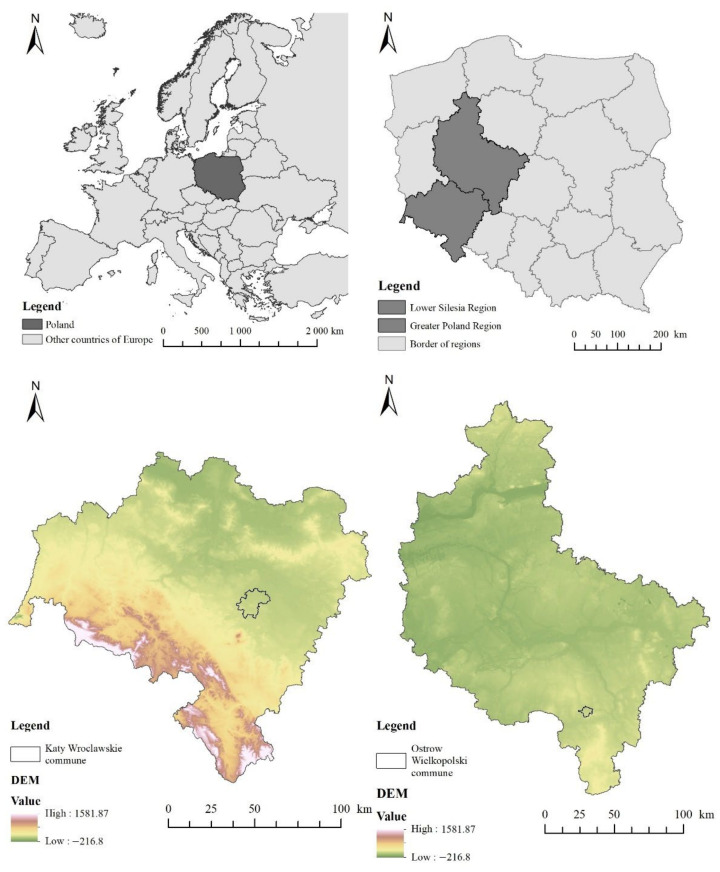
Location of communes on the background of Europe, Poland and the Lower Silesia region with the DEM base.

The areas of research consisted of two municipalities located in the southern part of Poland, Kąty Wrocławskie and Ostrów Wielkopolski. The Kąty Wrocławskie commune is located in the central part of the Lower Silesia region in Poland, whereas Ostrów Wielkopolski is located in in the southern part of the region called Greater Poland (Figure 1). The Kąty Wrocławskie commune borders with Wrocław, the capital of the region, and has an urban–rural character. The area of the commune is 17,700 ha. In 2019, the study area had 25,282 inhabitants and the population density per 1 km^2^ was 143 inhabitants. There are nature protection areas of different kinds located in the municipality: the Bystrzyca Valley Landscape Park, two Natura 2000 habitat areas, natural monuments and an ecological site. The commune lies in the geographical mesoregion Wrocław Plain and is located at an altitude of 120 to 220 m above sea level and has a low relief. The municipality is dominated by arable lands. Forests and meadows are located mainly in the Bystrzyca Valley Landscape Park. Road infrastructure is well developed. The A4 and A8 freeways run through the commune. Railroad line No. 247 from Wrocław to Jelenia Gora crossed the commune in the northern part.

The Ostrów Wielkopolski commune is located in the southern part of Greater Poland in Poland and has a municipal character. The surface area of the commune is 4190 ha. The population in 2019 was 71,947 and the density per 1 km^2^ is 1717 inhabitants. The municipality is located within a radius of 100 km from the center’s big cities, i.e., Wrocław, Poznan and Łodz. The commune lies in the geographical mesoregion Kaliska Upland at an altitude of 123 to 175 m above sea level and is characterized by a slight diversity of terrain relief. In the area of the commune, there is one retention reservoir which plays a recreational role. The dominant area of the city is occupied by urban development, and it is divided into multi-family and single-family residential complexes. A large area in the municipality is occupied by industrial or commercial units. A very well-developed rail network runs through the center from south to north. The commune also has a ring road, which is part of the S11 expressway, which will run from Kolobrzeg to Piekary Slaskie in the future.

The municipalities of Kąty Wrocławskie and Ostrów Wielkopolski are located in the southern part of Poland. Both municipalities are characterized by a flat terrain form and have good road or/and train connections. In the Kąty Wrocławskie commune, there are the A4 and A8 freeways, whereas the Ostrów Wielkopolski commune has a large railroad junction and ring road. The municipalities are located in two different provinces: Lower Silesia (Kąty Wrocławskie) and Greater Poland (Ostrów Wielkopolski). The first commune has an urban–rural character and the second one has an urban character. They differ in terms of size; Kąty Wrocławskie commune has an area of 17,700 ha, whereas Ostrów Wielkopolski has an area of 4190 ha. The population in the municipality of Kąty Wrocławskie is 25,282, whereas the population in Ostrów Wielkopolski is 71,947. The difference in the communes in terms of population density is noticeable; 12 times bigger in Ostrów Wielkopolski than in Kąty Wrocławskie, whereas Ostrów Wielkopolski has a population density of 1717 per km^2^. As of 2013, the population in the municipality of Ostrów Wielkopolski has been decreasing, in contrast to the municipality of Kąty Wrocławskie. In the Kąty Wrocławskie commune, there are forms of nature protection, which are absent in Ostrów Wielkopolski. The municipality of Kąty Wrocławskie borders a city of more than 100,000 people. The Ostrów Wielkopolski commune is at least 100 km away from other cities of more than 100 thousand people. Ostrów Wielkopolski lies 30 km from the Kalisz, forming the Kalisz–Ostrów agglomeration (Table 1)

### 2.2. Identification of Landscape Changes

#### 2.2.1. Research Procedure and Data

**Figure 2:**
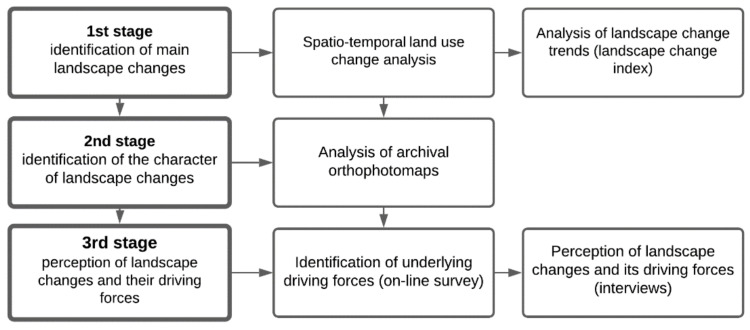
Scheme of landscape change assessment (source: own elaboration).

We analyzed changes in the landscape in the municipalities of Kąty Wrocławskie and Ostrów Wielkopolski in two time periods, 2006–2012 and 2012–2018, with the use of quantitative and qualitative methods. In the first stage of the study—identification of major changes in the landscape—we used quantitative methods defining area (in ha and %) of particular land-cover types within both case study areas which were collected on the basis of Corine Land Cover database for each of the analyzed periods—2006, 2012 and 2018. They were the basis for calculating the Landscape Change Index (LCI) developed by Krajewski [33]. This index illustrates the level of change in the landscape over a specified time period by aggregating changes within each land-cover type into a single value. Data were collected using GIS tools as recommended by González-Puente et al. [27] (a similar approach was used by Bauer et al., Brown and Weber, Jiang [34,35,36]). In order to assess the changes in the landscape identified in the first stage and visualized in the second stage by orthophoto maps, and to determine the driving forces that significantly contributed to the changes in the landscape during the 12-year study period, we used qualitative research methods typical of social research, including surveys of residents in the study areas and face-to-face semi-structured interviews that were designed to supplement knowledge regarding respondents’ perceptions of those changes. The scheme of research procedure is shown in (Figure 2).

Corine Land Cover (CLC) data were used to prepare the maps and databases from the Copernicus Land Monitoring Services website. In order to detail the areas where the land-use change occurred, archival spatial orthophoto maps from 2006, 2012 and 2018 were used. Data for the selected time period were obtained from the Central Office of Cartography and Geodesy, which were processed with the use of ArcMap software (Table 2).

#### 2.2.2. First Stage: Identification of the Main Landscape Changes

In the first step, we used the Corine Land Cover (CLC) database to create land-cover maps for the years 2006, 2012 and 2018. We used the landscape change index (LCI) developed by Krajewski [36] to examine the level of landscape change as the result of land-cover change in case study areas. The first step is to calculate the level of change in the percentage of each land-cover type. It was determined by the formula:

CA_i_ = 100 × (A_t+1_ − A_t_)/TA (1)

where CA_i_ represents changes in the percentage share of areas covered by each land-cover type (i) in relation to the total area of research (%); A_t+1_ represents the area covered with each type of land cover during the time interval t + 1 (ha); A_t_ represents the area covered with each type of land cover during the time interval t (ha), and TA represents the total research area (ha).

The landscape change index (LCI) measures, in a single numerical value, the level of change that occurs in a landscape as a result of changes in land cover. It was calculated with:

(2) $${\mathrm{LCI}}_{t}=\frac{1}{2}\times \sum_{i=1}^{n}\left|CA_{i}\right|$$

where LCI_t_ represents the landscape change index in each time interval and |CA_i_| represents the absolute value of change in percentage share of the areas covered by each land- cover type (i) in relation to the total research area.

We produced land-cover change maps for two time periods. We acquired free CLC (Corine Land Cover) vector data in the form of a geodatabase, whose accuracy is greater than 85%. The CLC land-cover change database was a separate product of a project prepared by the European Environment Agency in each period. Its objective was to record actual land-cover changes in Europe that covered more than 5 ha, were greater than 100 m in width, occurred in a discrete time period and were visible on satellite images. Raster data with land-cover changes were also available, but their quality was insufficient. The databases with changes between 2006 and 2012 differed when compared with the overlaid land-cover maps for 2006 and 2012; the same and similar were observed when the databases with changes in 2012–2018 were compared with the land-cover maps for 2012 and 2018. To determine the nature and extent of landscape transformations within municipal boundaries, data representing land-cover changes identified in the CLC database were processed using ArcMap 10.8.1. The land-cover changes revealed in the CLC database allowed for the creation of a classification of landscape changes. This classification includes six types of changes:

(1) conversion of arable land for urban development;
(2) conversions of arable land for commerce and services;
(3) conversion of meadow and pasture for urban development;
(4) conversion of arable land for communication areas;
(5) conversion of arable land for airport areas, and
(6) conversion of arable land for mineral extraction sites.

However, it should be emphasized that due to the boundaries arising from the specificity of the CLC database, the classification does not include land-cover change types with an area of less than 5 ha.

#### 2.2.3. Second Stage: Identification of the Character of Landscape Changes

In the second stage, we used the previously identified areas for a detailed analysis of the changes and characterization of newly created landscape elements. We analyzed the areas of landscape change identified at the first stage for both time periods using archival orthophoto maps from 2006, 2012 and 2018. It should be added, however, that the data collected in the CLC databases are not the same as the data presented on the orthophoto maps. This results from the adopted minimum mapping unit in the case of land-cover registration, which was 25 ha for surface phenomena and had a 100 m width for linear phenomena. The accuracy of guiding the borders of individual land-cover forms is 100 m. This allows for a detailed understanding of the nature of landscape changes, e.g., the type of newly constructed residential or commercial developments, and the identification of changes that are not large but are significant to the landscape. Those areas serve to present the areas of landscape change in the online survey (third stage).

#### 2.2.4. Third Stage: Identification of Driving Forces

##### Online Survey

**Figure 3:**
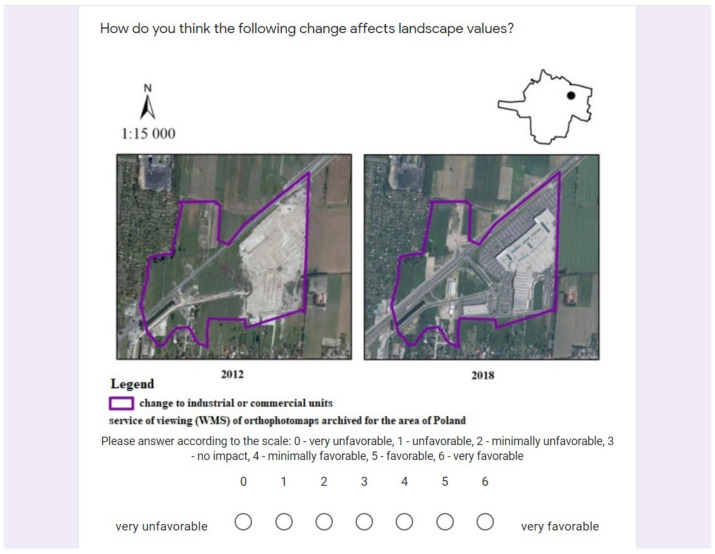
Interface of the online survey on the level of landscape changes, the influence on the landscape and the driving forces.

At the last stage, we identify the driving forces of landscape change using five main types of driving forces [4]. The forces were distinguished into socio-economic, political, technological, natural and cultural forces (a detailed list of driving forces is presented in Table A1). Driving forces were identified with the use of the online survey filled in between April and June 2021. Voluntary response sampling (type of non-probability sampling) was used for the online survey. The survey was posted on discussion groups about the study areas and on social media groups that had the name of the locality in their name. In addition, a link to the survey was distributed to organizations and associations that are related to the study area. The survey included individuals who, as volunteers, were willing to complete the survey of their own choice. Using such a sampling method does not allow us to estimate the size of the population that could have participated in the survey, therefore we cannot determine the sample size and consider it as representative sample. The survey consisted of three sections. The first section included background information related to gender, age, income level and the duration of living in the municipality. The second section included questions concerning the level of landscape changes in the years 2006–2018 (Figure 3). The third section presented areas of changes identified in the second stage and asked the respondents to evaluate those changes in terms of their influence on the landscape and the closed list of driving forces. For a better understanding of landscape changes, we conducted interviews with the inhabitants and asked about the perceived level of landscape changes.

##### Direct Interviews

**Figure A1:**
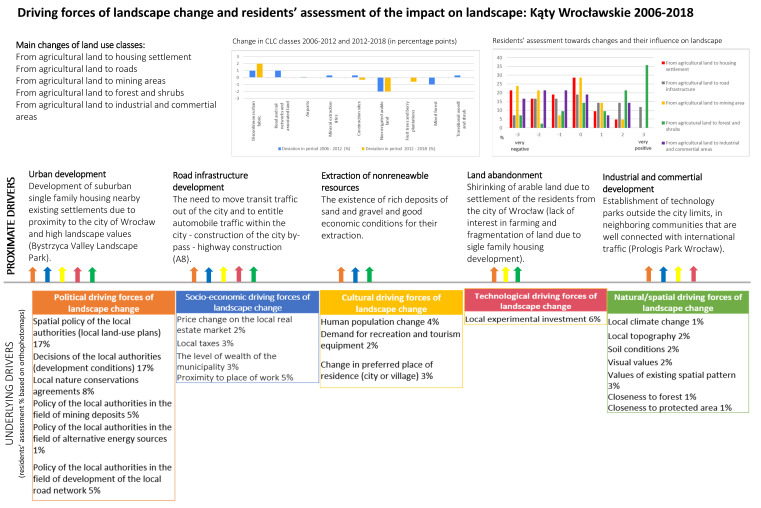
Guide to semi-structured interview.

The direct interviews were semi-structured and included the following topics: the intensity of landscape change, the drivers of landscape change, the impact on the residents, and their perception of those changes (Figure A1). Respondents were volunteers and did not receive any gratuity for their participation. The selection of people who were interviewed was based on one criterion—they should have lived in the research area for at least 15 years (have inhabitant status before 2006). The interviews were audio-recorded and transcribed verbatim. An interview guide was designed before the direct interviews with the aim of ensuring complete and consistent coverage of the themes under study in each interview. To preserve confidentiality, all participants were referred to by their participant numbers. We used narrative analysis to present respondents’ perception of landscape change. This method of analysis belongs to inductive methods of analyzing interview transcripts. It involves expert interpretation of individual interview respondents’ perception of landscape change. We used this type of qualitative data analysis to highlight important aspects of landscape change perception that will best resonate with the readers of the manuscript.

## 3. Results

### 3.1. Identification of the Main Landscape Changes

In the first stage, the level of landscape changes was assessed using the landscape change index (LCI) [36]. For both municipalities, the level of changes in the period of 2006–2012 was bigger than in the period of 2012–2018. For Kąty Wrocławskie, this index was 6 in the first analyzed period, with the value of 4.9 in the second period. The situation is similar in Ostrów Wielkopolski, where the landscape change index for the period 2006–2012 was 5, and for the period 2012–2018, it was only 2 (Table 3).

**Figure 4:**
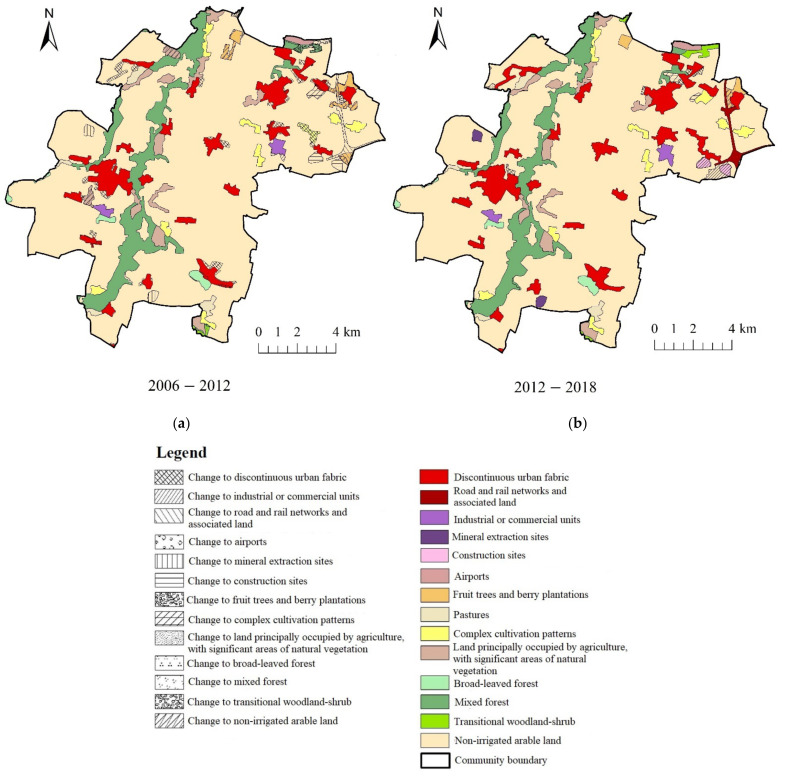
Location of landscape changes in the municipality of Kąty Wrocławskie: (**a**) 2006–2012; (**b**) 2012–2018.

**Figure 5:**
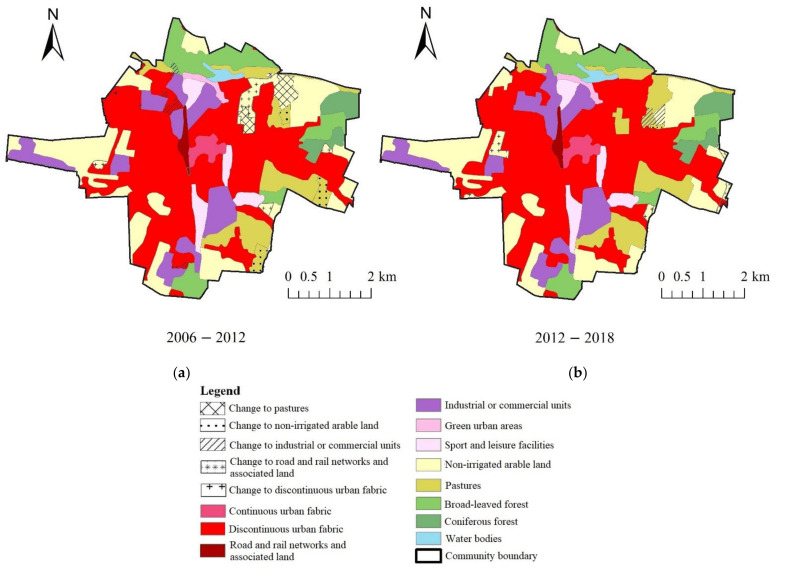
Location of landscape changes in the municipality of Ostrów Wielkopolski: (**a**) 2006–2012; (**b**) 2012–2018.

In both municipalities, the changes to the greatest extent concerned the transformation from non-irrigated arable land to discontinuous urban fabric (Table 4). In the years of 2006–2012 in the municipality of Kąty Wrocławskie, the amount of non-irrigated arable land and mixed forest decreased by less than 2% (Table 4, Figure 4), whereas area of other land-cover types increased—discontinuous urban fabric (+1.21%), road and rail networks and the associated land (+0.66%), airport areas (+0.05%), mineral extraction sites (+0.3%), construction sites (+0.32%) and transitional woodland shrub (+0.41%) increased. In the years of 2012–2018, the area of discontinuous urban fabric increased by 1.7%, industrial and commercial units increased by 0.64%, while construction sites (−0.32%), non-irrigated arable land (−2.16%) and fruit trees and berry plantations (−0.16%) decreased. In the municipality of Ostrów Wielkopolski in the years of 2006–2012, the area of discontinuous urban fabric (+1.12%), industrial or commercial units (+0.72%) and pastures (+0.1) increased, whereas non-irrigated arable land decreased by 1.87% (Table 5, Figure 5). In the years of 2012–2018, the discontinuous urban fabric continued to increase by 1.03% and non-irrigated arable land decreased by 1.58%.

### 3.2. Identification of the Character of Landscape Changes

At the second stage of the analysis, we chose one example for each type of landscape change in both case study areas. As a result, eight areas of landscape change were presented, first in the municipality of Kąty Wrocławskie and then in Ostrów Wielkopolski. Below, we illustrated five examples of changes in the municipality of Kąty Wrocławskie occurring in the first examined period from 2006 to 2012. For the municipality of Ostrów Wielkopolski, three areas were presented: the first of them concerned the years between 2006 and 2012 and the next two, the years between 2012 and 2018. The examples of changes are accompanied by the perceived impact of the change on the landscape from the survey (third stage).

**Figure 6:**
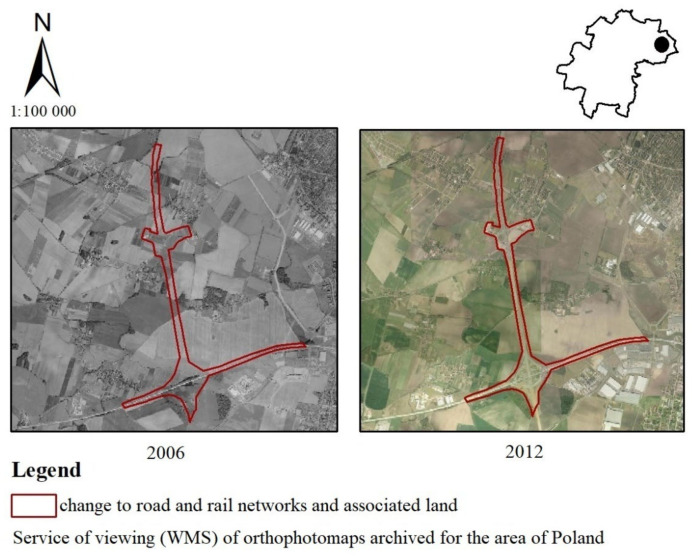
Change from non-irrigated arable land to road and rail networks and associated land in the Kąty Wrocławskie commune.

Numerous changes have been observed in the north-eastern part of the Katy Wrocławskie commune (Figure 6). In 2011, the A8 Wrocław freeway was built in order to relieve the traffic routes of Wrocław. At the same time, the Wrocław West and Wrocław South interchanges were created. The Wrocław South interchange located near Nowa Wies Wrocławska intersects with the A4 freeway. According to a questionnaire addressed to the inhabitants of the Kąty Wrocławskie commune, the answers related to this change were divided. Some respondents said that the change had no impact on landscape values (19%). The rest of the residents were divided into favorable (12%—very positive, 14%—positive, 14%—moderately positive) and unfavorable impact (7%—very negative, 17%—negative, 17% moderately negative).

**Figure 7:**
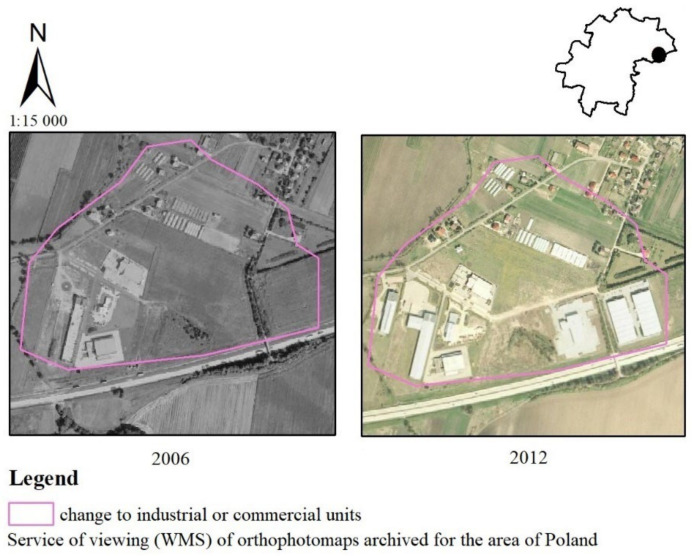
Change from non-irrigated arable land to industrial or commercial units in the Kąty Wrocławskie commune.

Between 2006 and 2012, new areas of industrial or commercial units appeared on arable land in the southern part of the municipality near the A4 freeway junction (Figure 7). These areas in the following period (2012–2018) were also expanded with more warehouses. According to the survey responses, the vast majority of residents felt that the above change had an adverse effect on scenic values (17—very negative, 21%—negative, 21%—moderately negative). Some of the respondents also felt that the change had no impact on landscape values (19%). Minority has seen this change as having a favorable effect on the landscape (7%—moderately positive, 14%—positive, 0%—very positive).

**Figure 8:**
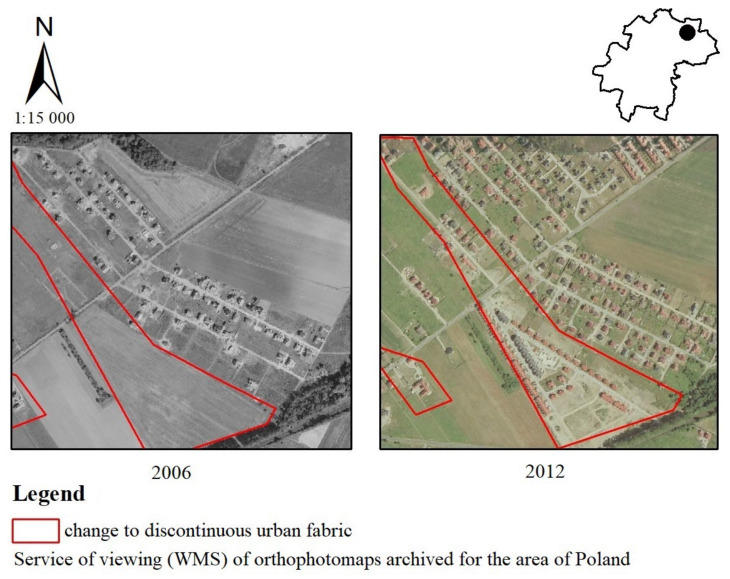
Change from non-irrigated arable land to discontinuous urban fabric in the Kąty Wrocławskie commune.

The most frequent changes in the municipality of Kąty Wrocławskie concerned the transformation of non-irrigated arable land into new discontinuous urban fabric (Figure 8). Such a change in years is shown above, in Smolec in 2006–2012. Perpendicular to Chłopska Street, new single-family and terraced houses were built. Almost half of the residents surveyed felt that the above change had a negative impact on landscape values. A much smaller number of people considered the change to be positive, whereas a smaller half said the change had no effect on landscape values (very negative—24%, negative—21%); 7% evaluated this change as moderately negative. Nobody valuated this change as having a very positive impact on landscape. A much smaller number of people considered the change to be positive (5%) and moderately positive (14%), whereas 29% said the change had no effect on landscape values.

**Figure 9:**
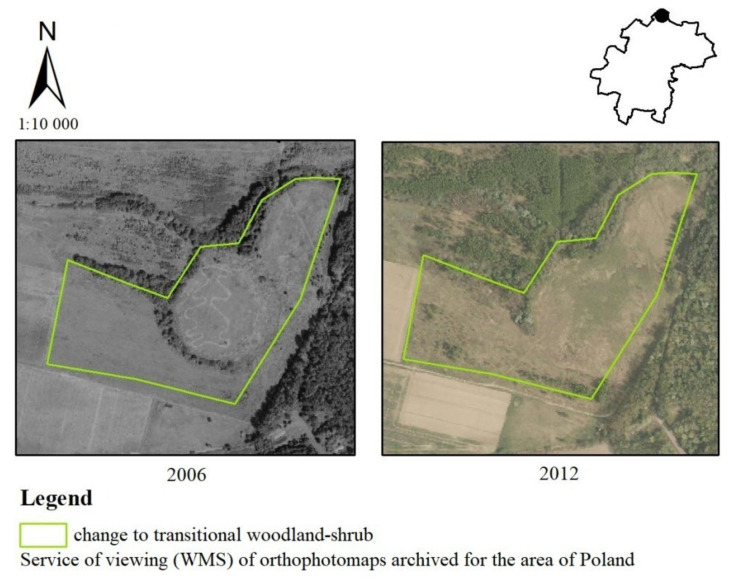
Change from non-irrigated arable land to transitional woodland-shrub in the Kąty Wrocławskie commune.

In the analysis of archival orthophotos shown in Figure 9, an increase in the area of forest and shrub vegetation in the state of change was evident. It occurred in the years 2006–2012 in the locality of Samotwór in the northern part of the Kąty Wrocławskie commune. If we take into account the answers of the residents of the Kąty Wrocławskie commune, the above-mentioned change had a positive impact on the landscape (36%—very positive, 21%—positive, 10%—moderately positive). There were also a few respondents who said it had no impact (14%) or an unfavorable impact (7%—very negative, 2%—negative, 10%—moderately negative).

**Figure 10:**
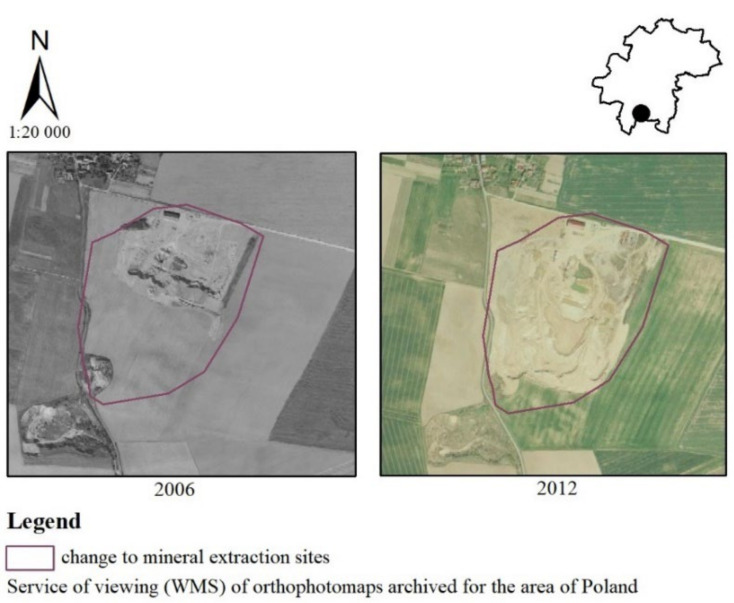
Change from non-irrigated arable land to mineral extraction sites in the Kąty Wrocławskie commune.

The example of the transformation of non-irrigated arable land to mineral extraction sites in the southern part of the municipality in the years 2006–2012 is shown in Figure 10. A natural aggregate mine “Siedlakowice I” was established in the village of Zachowice. The main deposits mined in this area are sands and gravels. According to half of the votes in the survey, the inhabitants considered the above change as unfavorable (24%—very negative, 21%—negative, 7%—moderately negative). Additionally, some people considered the change as having no impact on landscape values (29%). The vast minority answered that it was beneficial (5%—positive, 14%—moderately positive).

**Figure 11:**
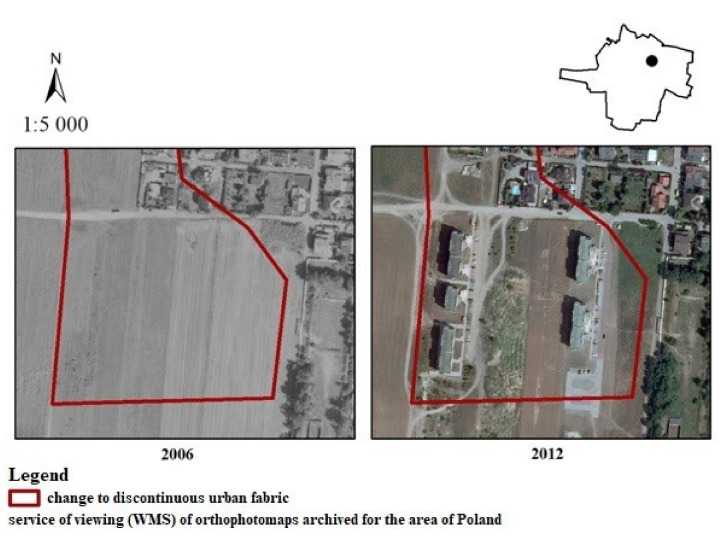
Change from non-irrigated arable land to discontinuous urban fabric in the city of Ostrów Wielkopolski.

In the Ostrów Wielkopolski commune, three types of landscape changes were presented. First area is located in the northern part of the municipality. It is a part of the Wenecja settlement (Figure 11), which is the largest in terms of area in the municipality. There has been a change from arable land to discontinuous urban fabric, which is connected with the expansion of the Wenecja settlement. Multi-family housing was built there. The area is located close to the city center and is well connected with it. It is located in close proximity to a sports and recreation area with a retention reservoir adapted for recreational purposes.

According to the questionnaire addressed to the residents of the Ostrów Wielkopolski municipality, the answers related to this change were divided. A large majority of the population responded that the above change had a negative impact on landscape values (2%—very negative, 17%—negative, 29%—moderately negative). The rest of the people said it had no influence (17%) or had a positive impact (5%—very positive, 17%—positive, 14%—moderately positive).

**Figure 12:**
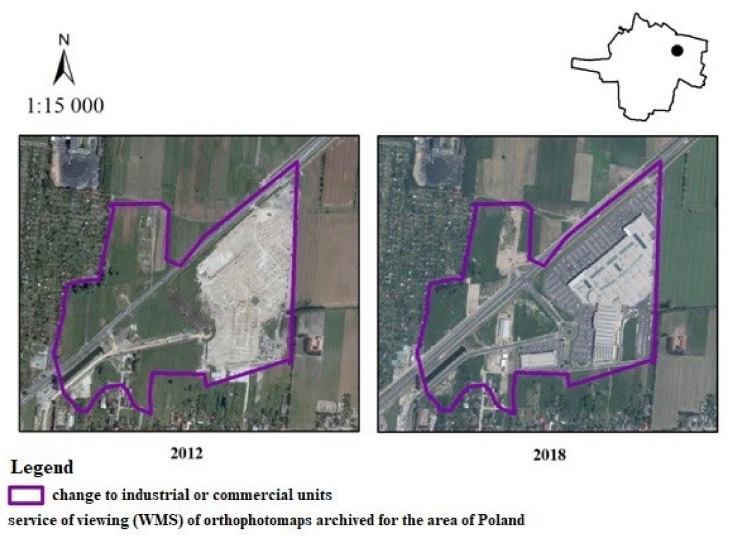
Change from non-irrigated arable land to industrial or commercial units in the city of Ostrów Wielkopolski.

The next area shows the formation of a commercial and service unit on the outskirts of the city (Figure 12), which occupied land previously used as meadows, pastures and arable land. In the western part of the municipality, in the Krepa settlement, a gallery was built. The commune signed an agreement with the developer, in which the developer undertook to bear the costs of construction of a collective road running next to the shopping center which improved communication. It also caused the development of investments nearby, namely the construction of a petrol station and an additional shopping passage. According to the survey responses, the public were again divided. Some of those surveyed felt that the change had a beneficial impact on scenic values (10%—very positive, 12%—positive, 17%—moderately positive), whereas some felt that it had an adverse impact (12%—very negative, 17%—negative, 17%—moderately negative). Additionally, there was a group of people for whom the change had no impact (17%).

**Figure 13:**
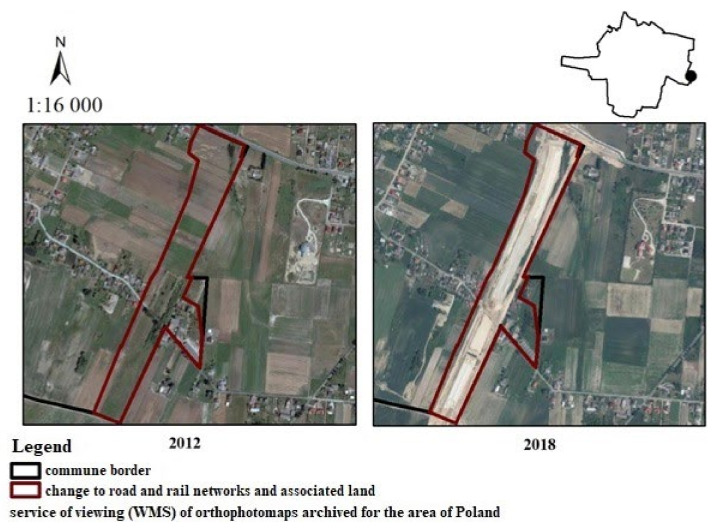
Change from non-irrigated arable land to road and rail networks and associated land in the city of Ostrów Wielkopolski.

The next area is the ring road of Ostrów Wielkopolski. It is located in the eastern part of the commune and is surrounded from most sides by arable lands or forests. Previously, the area was used as arable land which was transformed into the ring road in July 2017 (Figure 13). The Ostrów Wielkopolski bypass is part of the S11 expressway, which is to run from Kolobrzeg to Piekary Slaskie in the future. It is designed to take transit traffic out of the city center. If we take into account the responses of the residents of the municipality of Ostrow Wielkopolski, as with the above changes, there was no uniform response. The community was divided; half of the people said that the change had a positive impact on landscape values: 2%—very negative, 14%—negative, 17%—moderately negative), whereas there were also responses saying it had no impact (21%) or an unfavorable impact (12%—very positive, 17%—positive, 12%—moderately positive).

### 3.3. Identification of Driving Forces

#### 3.3.1. The Impact of the Changes on Landscape Perception

The impact of the changes on landscape perception was evaluated by the respondents in both municipalities. We collected 84 responses from volunteer respondents in both municipalities. The demographic characteristics of survey participants are shown in Table 6.

Table 7 shows the relation between age groups of the survey respondents in relation to the census data in both case study areas. In Ostrów Wielkopolski, the group of young adults (21–30 years old) were strongly overrepresented, and adults (31–40 years old) as well as older adults (51–60 years old) were overrepresented, while other groups were underrepresented. In Kąty Wrocławskie, adults in the age of 31–40 and 41–50 were overrepresented and other age groups were underrepresented. Due to the low representativeness of the survey respondents, we do not draw conclusions about the relation between age and perception of landscape changes and their driving forces. We are also aware that the opinions presented in the survey might be more relevant for some age groups and less relevant for others. The over representativeness of adults and young adults might be caused by the use of the sampling method.

**Figure A2:**
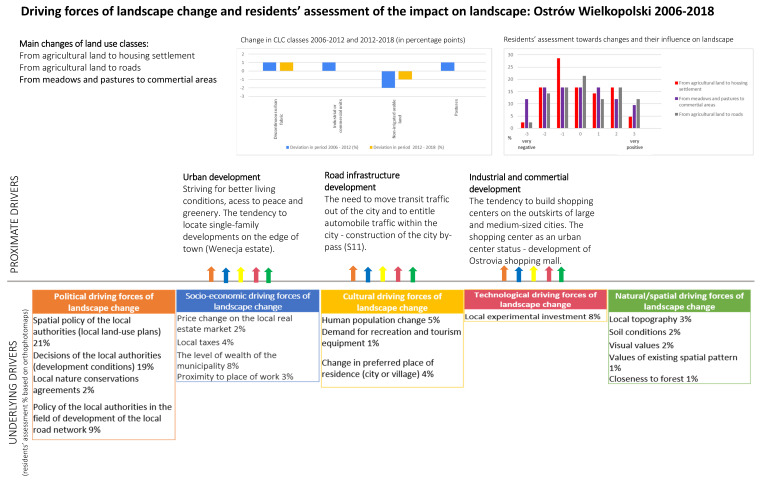
The full list of identified driving forces of landscape change and residents’ assessment of the impact of these drivers on the landscape in years 2006–2018 in Kąty Wrocławskie commune and Ostrów Wielkopolski city.

In the municipality of Kąty Wrocławskie, the changes from agricultural land to housing were evaluated as generally neutral to very negative, which is similar to the evaluation of the change from agricultural land to mining area. The respondents’ perceptions of the change from agricultural land to road infrastructure ranged from very negative to positive, similar to the change from agricultural land to industrial and commercial areas. The change from agricultural land to forest and shrubs was evaluated rather positively. In the municipality of Ostrów Wielkopolski, the perceptions of all types of changes (from agricultural land to housing, from meadows and pastures to commercial areas, and from agricultural land to roads) ranged from very positive to very negative. A detailed comparison is presented in Figure A2.

#### 3.3.2. Proximate and Underlying Driving Forces

In the municipality of Kąty Wrocławskie, we identified five proximate drives: urban development, road infrastructure development, the extraction of non-renewable resources, and land abandonment, and related them to underlying drivers indicated by the respondents. In the municipality of Ostrów Wielkopolski, we identified three proximate drivers: urban development, road infrastructure development, and industrial and commercial development, as well as accompanying proximate drivers identified by the respondents. A detailed elaboration of the proximate and underlying driving forces in both research areas is shown in Figure A2.

#### 3.3.3. Interview Analysis

##### Ostrów Wielkopolski

We conducted seven interviews in the municipality of Ostrów Wielkopolski. Two respondents lived in the municipality for a period between 15 and 30 years (28%) and five respondents for more than 30 years (72%). A total of 43% of the respondents were between 21 and 30 years old, 43% between 51 and 60 years old, and 14% over 60 years old. A total of 67% of respondents perceived the landscape changes in the municipality as medium and 43% as high. We discuss citizens’ perceptions towards different types of landscape changes.

- Urban development

The interviews revealed that the change from agricultural land to housing is often mentioned by the participants:

“*The biggest change is the emergence of residential development; practically at every step, a new house or block of flats is being built.*”

(Interviewee 4, aged 54)

“*The biggest changes were the expansion of multi-family and single-family housing developments on the outskirts of the city. I think this was caused by changes in land use, or favorable purchase prices and costs to convert agricultural land for development.*”

(Interviewee 5, aged 23)

This type of change is generally perceived positively:

“*On the estate where I live, many new single-family houses are being built. But for me, it is a positive change.*”

(Interviewee 2, aged 60)

However, the lack of greenery in the city was also noticed by the respondents:

“*A new block of flats was built next to my block, in a place where nobody expected it. It fits in nicely, but there could be some place with greenery there because there is not enough of it in the city.*”

(Interviewee 1, aged 57)

- Industrial and commercial development

The change from meadows and pastures to commercial areas (in this case, the construction of the shopping mall) was generally perceived positively in terms of new job opportunities:

“*The gallery and the ring road are big investments. I think they brought a lot of conveniences and jobs to the city, as far as the gallery is concerned.*”

(Interviewee 2, aged 60)

The accompanying infrastructure was also generally perceived positively:

“*A positive change, for sure, was the construction of the gallery. Thanks to it, a new road was built which connected my place of residence with the exit to, for example, the main street in the city and the road to Kalisz.*”

(Interviewee 3, aged 61)

However, the impact of the new shopping mall on the little shops in the city center was also noticed by the respondents living in the city center:

“*The only disadvantage is that the construction of the mall eliminates many of the stores in the city center, so you have to drive to the mall.”*

(Interviewee 1, aged 57)

- Development of road infrastructure

The change from agricultural land to roads was perceived differently depending on the place of residence; it was perceived positively by the people living in the city center:

“*For me, an important change was the construction of the ring road. I think it was needed here because it reduced traffic jams in the city and accidents. I live by the main road in the city. Thanks to the ring road, the noise was reduced. Previously, it was unbearable with open windows.*”

(Interviewee 1, aged 57)

It was perceived negatively by the citizens living in the outskirts of the city :

“*For me, the ring road is particularly burdensome as I can hear quite a lot of noise.*”

(Interviewee 3, aged 61)

Additionally, the respondents noticed the need for more recreation areas within the city:

“*I miss new bicycle paths and places with greenery in the city as well as sports recreation places.*”

(Interviewee 4, aged 54)

##### Kąty Wrocławskie

We conducted four interviews in the municipality of Kąty Wrocławskie. The participants lived in the case study area for a rather long period of time (between 23 and 75 years). All participants live in the town of Kąty Wrocławskie. The changes within the municipality were perceived as medium by one respondent, and as high by the rest of the participants (three people). We discuss the citizens’ perceptions towards different types of landscape changes below.

- Settlement growth

The change from agricultural land to housing is generally perceived negatively by the inhabitants due to the chaotic planning and high housing density. In particular, the new development in Smolec is criticized:

“*Smolec is an example of I don’t know what, it’s just a tragedy, for me. It’s a tragedy. How it’s built there, I don’t know if anyone has oversight over it at all.*”

(Interviewee 1, aged 75)

“*They made such a kolkhoz. Around Smolec and there, in the direction of Kąty Wrocławskie, Wrocław is simply moving with the housing industry.*”

(Interviewee 4, aged 75)

However, the new housing is also perceived as a positive development:

“*The fact that buildings are being erected in the so-called utility fields is a good thing. (…) No development has a negative effect; on the contrary, its absence would have a negative effect.*”

(Interviewee 2, aged 23)

Probably due to the high intensity of changes, the inhabitants start to wonder why those changes happen and they find an economical reason:

“*Do I know, what drew people here to these Kąty Wrocławskie is a wonder. It just makes me wonder. If I didn’t have to live here, I wouldn’t live in Kąty Wrocławskie. That’s why I always say to my husband, what do these people see in these parts, nothing is happening here. The swimming pool is closed all the time, constantly, there is no alternative for the youth. I myself would like to know what attracts them here. Maybe the prices, maybe it’s cheaper. I have the impression that this is the only thing that can attract people here. The proximity of the big city of Wrocław, people commute somewhere there.*”

(Interviewee 3, aged 47)

Another reason they find is seeking peace:

“*People run away from Wrocław to have peace and quiet. Although, I don’t know if it’s peaceful in Smolec, where one person sits on top of another.*”

(Interviewee 4, aged 54)

- Development of road infrastructure

The road infrastructure and its development are of great importance for the inhabitants. The ring road around Wrocław (A8) is perceived positively and as a necessity:

“*If there was no bypass, we wouldn’t be talking about it because Wrocław would be blocked for good.*”

(Interviewee 4, aged 54)

The inhabitants also see the railway development as a positive change and an alternative to driving:

“*The A4 freeway is supposed to be the axis of development, but there is a problem with it because something is happening all the time, it is too narrow, too small, transit transport jams. A lot has been put on the railroad and it has been successful. This is really a proven element of railroads.*”

(Interviewee 4, aged 54)

The inhabitants point out the fact that traffic on the Wrocław bypass is often stopped because of accidents and they see the need to build a bypass for Kąty Wrocławskie:

“*We, the residents of Smolec, would very much like there to be a ring road passing by Kąty and Kostomłoty. This is transit. If anything happens on the highway, we all feel it and it’s simply a tragedy.*”

(Interviewee 1, aged 75)

“*A bypass of Kąty Wrocławskie would be absolutely necessary. Something like this would be useful, which would cause when A4 is blocked because there are accidents, Kąty Wrocławskie is a town in which cars drive all the time. They drive through the main street, there is also a school there. It is a nuisance for the residents.*”

(Interviewee 2, aged 23)

The inhabitants would also like to have more bicycle paths and adaptations for the disabled. The restoration of the city park was also noticed and pointed out as a positive change for the city. The respondents do not refer to the change from agricultural land to mining, industrial and commercial areas, or to forest and shrubs.

## 4. Discussion

Landscape changes were more intense in the period of 2006–2012 than between the years of 2012–2018. Both municipalities experienced a decrease in agricultural land and a simultaneous increase in housing development. There is a large agreement between perceived and actual land-cover change which goes in line with the result of Twongyirwe et al. [30]. This might confirm that the residents are aware of the landscape change and, concerning the theory of landscape perception, that the changed landscape might also affect them [37].

The analysis of the types of transformations of particular land-cover elements identified in both study areas, and the main causes of landscape transformations indicated by the respondents for the areas presented in the survey, made it possible to indicate 5 main landscape change processes taking place in the landscape in the municipality of Kąty Wrocławskie—urban development, road infrastructure development, the extraction of nonrenewable resources, land abandonment and industrial and commercial development, and 3 processes in Ostrów Wielkopolski—urban development, road infrastructure development and industrial and commercial development. These processes can be called proximate driving forces—they are the direct driving force of changes in the landscape, in contrast to the underlying driving forces, which are the cultural, economic or technological background, thus providing a basis for the changes. Summaries of landscape change processes together with the major types of land-cover transformations and the underlying driving forces behind these changes are presented in Figure A2. The perceptions of the impact of urban development on the landscape in the urban municipality range from very negative to very positive and the perceptions of the impact of urban development on the urban–rural landscape are rather negative or neutral. The interviews revealed that urban development in Ostrów Wielkopolski is described rather positively by citizens; however, they also point out the lack of green spaces. On the other hand, urban development in Kąty Wrocławskie is perceived as being very negative. This result confirms a low social acceptance of landscape change on agricultural land which goes in line with the study of Bevk and Golobič [24]. They studied the perception of landscapes changed by solar power plants. Their results showed that on-ground solar power installations were valued differently based on the type of land they occupy, with degraded land being the most accepted and farming land being the least accepted. The setting is probably not the only relevant factor here but also the way in which new development is carried out. Citizens complain because of chaotic planning, and a lack of privacy and noise. Due to a high intensity of urban development, the interviewees tended to consider the drivers of landscape change, and listed lower prices for houses and seeking peace and quiet as possible reasons for moving to the municipality of Kąty Wrocławskie.

Road infrastructure development is perceived rather positively in the urban setting and the perceptions ranged from very negative to very positive in the urban–rural landscape. According to the interviews, the perception of the new road in Ostrów Wielkopolski depended on its effect on the individual (better communication possibilities but also the exposition to noise). Although the development of road infrastructure was perceived as an absolute necessity by the citizens of Kąty Wrocławskie, they also expressed the need for further road infrastructure development. This also underlines the strong functional relationship between the suburban area and the city of Wrocław. The influence on the landscape of industrial and commercial development is perceived very diversely in both municipalities. The interviews show that the new commercial area in Ostrów Wielkopolski tends to be identified with economic development; however, it was also noted that due to the new shopping mall, many small shops in the city center disappeared. Land abandonment in the suburban area is perceived very positively, which might be caused by the presence of a new forest that is associated with closeness to nature by suburban dwellers. The extraction of nonrenewable resources is perceived very negatively in the municipality of Kąty Wrocławskie.

In both municipalities, the respondents pointed out the political driving forces of landscape change as key underlying drivers (especially the spatial policy of the local authorities expressed in local land-use plans and the decisions of the local authorities included in development conditions), although we assume that in the case of the municipality of Kąty Wrocławskie, the proximity to Wrocław combined with low housing prices could be of great importance. This result confirms that the identification of driving forces by residents does not always reflect the whole situation. Similar results are shown in the study by Aretano et al. [29] where the residents of Mediterranean islands do not consider tourism as the driving force of the urban sprawl that is the main cause of landscape change, probably because local residents perceive it as the main motor of economic development.

Due to a relatively low number of participants, we could not draw conclusions about the influence of age on the perception of landscape change. We are aware that a possible effect of spatial discounting understood here as the uneven distribution of opinions (some areas of the municipalities are represented better than others) is that it might influence our results [38]. Therefore, we only draw conclusions for the areas which are covered by the interview participants. Significant is the small number of direct interviews that were conducted with 11 people who also responded to the survey. It was assumed that these would be people living in the study area for at least 15 years, since the research covered the period from 2006. Although the sample was selected to include representatives of different age ranges and a comparable number of men and women, we realize that these results do not reflect the perception of changes in the landscape of the entire society. The direct interviews should therefore be treated as supplementary and more detailed material for the survey research. We believe that this approach can be improved and applied in different case study areas.

## 5. Conclusions

In this paper, we identified landscape changes and their driving forces in urban and urban–rural settings. We concluded that landscape changes were more intense in the period 2006–2012 than in the years 2012–2018. Both municipalities experienced a decrease in agricultural land and a simultaneous increase in housing development, and identified urban development, road infrastructure development, and industrial and commercial development as key landscape change processes. We identified 13 landscape transformation types in Kąty Wrocławskie commune and 5 types in Ostrów Wielkopolski city. In both municipalities, the changes to the greatest extent concerned the transformation from non-irrigated arable land to discontinuous urban fabric. Generally, the changes from agricultural land to housing as well as the evaluation of the change from agricultural land to mining area were evaluated as neutral to very negative. The change from agricultural land to road infrastructure and the change from agricultural land to industrial and commercial areas is perceived from very negative to positive. The change from agricultural land to forest and shrubs was evaluated as a positive change. Citizens’ perceptions concerning key landscape changes in both municipalities differed depending on the context, level of changes, and the way this process was planned and implemented. Residents in both study areas identified driving forces from each of five groups, including political, natural, technological, socioeconomic, and cultural forces. The most frequently identified driving forces for landscape change were political forces, among which, by far the most frequently identified by residents were local authorities’ spatial policies regarding land-use plans and building decisions. Other important political drivers in Kąty Wrocławskie were legal regulations regarding the functioning of protected areas and the local government’s policy on mining areas, as well as the proximity of workplaces (the municipality directly neighbors with Wrocław) from the group of socioeconomic forces. In Ostrów Wielkopolski, another driving force included in the group of political forces was the local government’s policy on road network expansion and local experimental investments included in the technological forces of landscape change. Future landscape planning should consider citizens’ approaches towards landscape change and implement their statements to achieve better societal approval and a higher quality of life.

## Figures and Tables

**Table 1:** Comparison of data for the municipalities of Kąty Wrocławskie and Ostrów Wielkopolski.

Municipality	Region	Area (ha)	Population	Density per 1 km^2^	Type of Commune	Main Landscape Features
Kąty Wrocławskie	Lower Silesia	17,700	25,282	143	urban–rural	Flat area bordering Wrocław with a predominantly agricultural landscape. Built-up areas prevail in the central part of the commune in the city and in the north-eastern part of the commune.
Ostrów Wielkopolski	Greater Poland	4190	71,947	1717	urban	Flat area with a historical city center, recreational areas and new housing estates in the northern part, and a shopping mall and transport hub in the north-eastern part.

**Table 2:** Data used for analysis for Kąty Wrocławskie and Ostrów Wielkopolski municipalities.

Data Used for Analysis	Type of Data	Year	Database	Source
Corine Land Cover	Shapefile	2006, 2012, 2018	CLC2006, CLC2012, CLC2018	Copernicus Land Monitoring
Standard archive orthophoto map	Raster with georeference	2006, 2012, 2018	Polish geoportal, Infrastructure for Spatial Information	Central Office of Cartography and Geodesy

**Table 3:** The percentage of deviation for types of land cover in the municipalities of Kąty Wrocławskie and Ostrów Wielkopolski in the analyzed periods.

Corine Land Cover Classes	Kąty Wrocławskie		Ostrów Wielkopolski	
	Deviation in Period 2006–2012 (%)	Deviation in Period 2012–2018 (%)	Deviation in Period 2006–2012 (%)	Deviation in Period 2012–2018 (%)
Discontinuous urban fabric	1	2	1	1
Industrial or commercial units	0	0	1	0
Road and rail networks and associated land	1	0	0	0
Airport area	0.1	0	0	0
Mineral extraction sites	0.3	0	0	0
Construction sites	0.3	−0.3	0	0
Non-irrigated arable land	−2	−2	−2	−1
Fruit trees and berry plantations	0	−0.6	0	0
Pastures	0	0	1	0
Mixed forest	−1	0	0	0
Transitional woodland shrub	0.3	0	0	0
Landscape Change Index	6	4.9	5	2

**Table 4:** Area of each land-cover class in different time periods in the Kąty Wrocławskie commune.

CLC Code	CLC Class	Kąty Wrocławskie					
		2006		2012		2018	
		Area (ha)	Area (%)	Area (ha)	Area (%)	Area (ha)	Area (%)
1.1.2.	Discontinuous urban fabric	1181.17	6.69	1395.49	7.90	1695.40	9.60
1.2.1.	Industrial or commercial units	88.70	0.5	98.16	0.55	210.81	1.19
1.2.2.	Road and rail networks and associated land	-	-	117.03	0.66	117.03	0.66
1.2.4.	Airport area	38.23	0.22	47.44	0.27	47.44	0.27
1.3.1.	Mineral extraction sites	-		53.12	0.30	53.12	0.30
1.3.3.	Construction sites			57.32	0.32	-	
2.1.1.	Non-irrigated arable land	12,882.13	72.92	12,557.25	71.09	12,177.13	68.93
2.2.2.	Fruit trees and berry plantations	188.19	1.06	89.79	0.51	64.42	0.36
2.3.1.	Pastures	125.13	0.71	125.13	0.71	125.13	0.71
2.4.2.	Complex cultivation patterns	299.47	1.7	298.97	1.69	339.56	1.92
2.4.3.	Land principally occupied by agriculture with significant areas of natural vegetation	513.77	2.91	452.62	2.56	460.16	2.60
3.1.3.	Broad-leaved forest	105.17	0.6	110.67	0.63	110.67	0.63
3.1.3.	Mixed forest	2219.04	12.56	2182.00	12.35	2184.13	12.36
3.2.4.	Transitional woodland shrub	24.32	0.14	80.28	0.45	80.28	0.45

**Table 5:** Area of each land-cover class in different time periods in the Ostrów Wielkopolski city.

CLC Code	CLC Class	Ostrów Wielkopolski					
		2006		2012		2018	
		Area (ha)	Area (%)	Area (ha)	Area (%)	Area (ha)	Area (%)
1.1.1.	Continuous urban fabric	57.76	1.38	57.76	1.38	57.76	1.38
1.1.2.	Discontinuous urban fabric	1728.00	41.24	1775.00	42.36	1818.00	43.39
1.2.1.	Industrial or commercial units	411.63	9.82	440.00	10.50	470.00	11.22
1.2.2.	Road and rail networks and associated land	32.25	0.77	31.00	0.74	46.00	1.10
1.4.1.	Green urban areas	25.62	0.61	25.62	0.61	25.62	0.61
1.4.2.	Sport and leisure facilities	141.34	3.37	141.34	3.37	141.34	3.37
2.1.1.	Non-irrigated arable land	884.57	21.11	806.00	19.24	740.00	17.66
2.3.1.	Pastures	396.09	9.45	400.00	9.55	379.00	9.05
3.1.3.	Broad-leaved forest	361.74	8.63	361.74	8.63	361.74	8.63
3.1.2.	Coniferous forest	122.99	2.94	122.99	2.94	122.99	2.94
5.1.2.	Water bodies	28.30	0.68	28.30	0.68	28.30	0.68

**Table 6:** Demographic characteristics of the survey participants.

Variable	Ostrów Wielkopolski		Kąty Wrocławskie	
	No	%	No	%
Duration of living				
1–5 years	1	2	1	2
5–15 years	2	5	9	21
15–30 years	20	48	11	26
More than 30 years	19	45	21	50
Education				
Primary	1	2	2	5
High school	18	43	7	17
Higher	23	55	26	62
Income level				
Lack of earnings	2	5	4	10
Low (around minimum wage)	6	14	5	13
Medium (below the national average)	22	52	9	23
High (over the national average)	12	29	20	52
Age				
Below 20	1	2	1	2
21–30	20	48	3	7
31–40	9	21	11	26
41–50	2	5	9	21
51–60	9	21	2	5
Over 60	1	2	2	5
Assessment of the level of landscape change				
Low	9	21	6	14
Medium	18	43	12	29
High	15	36	24	57

**Table 7:** Age groups of survey respondents in relation to the census data for Ostrów Wielkopolski and Kąty Wrocławskie.

Age Group	Ostrów Wielkopolski		Kąty Wrocławskie	
	Respondents (%)	Census Data (%)	Respondents (%)	Census Data (%)
Below 20	3	16	2	22
21–30	48	9	10	10
31–40	21	14	40	16
41–50	5	13	33	16
51–60	21	11	7	10
Over 60	2	37	7	26

## Data Availability

In this research we used publicly open data: Corine Land Cover (CLC) from the Copernicus Land Monitoring Services website and archival spatial orthophoto maps for years 2006, 2012 and 2018 from the Central Office of Cartography and Geodesy, accessed via website www.geoportal.gov.pl, accessed on 30 December 2021. All other data is stored in the archive of the Institute of Spatial Management, Wroclaw University of Environmental and Fife Sciences.

## Appendix A

The full list of possible driving forces of landscape change that were used in the surveys.

**Table A1:** The list of possible driving forces of landscape change in Poland on local level.

Types of driving force of landscape change
Political driving forces of landscape change
Spatial policy of the local authorities (local land-use plans)
Decisions of the local authorities (development conditions)
Local nature conservation agreements
Policy of the local authorities in the field of mining deposits
Policy of the local authorities in the field of alternative energy sources
Policy of the local authorities in the field of development of the local road network
Socio-economic driving forces of landscape change
Lower price levels in the local real estate market
Local taxes
The level of wealth of the municipality
Low unemployment in the commune
Proximity to place of work
Cultural driving forces of landscape change
Human population change
Demand for recreation and tourism equipment
Change in preferred place of residence (city or village)
The need to build second homes in recreation areas
Technological driving forces of landscape change
Local experimental investment
Natural/spatial driving forces of landscape change
Local climate change
Local extreme phenomena such as landslides, storms, etc.
Local topography
Soil conditions
Visual values
Values of existing spatial pattern
Closeness to the forest
Closeness to the protected area
